# The predictive value of MUC5AC levels in the sputum of children with *Mycoplasma pneumoniae* pneumonia treated with fiberbronchoscopy

**DOI:** 10.1128/spectrum.00103-25

**Published:** 2025-07-02

**Authors:** Xiao-qing Jian, Miao Li, Xiao-yan Wang

**Affiliations:** 1 Department of Pediatrics, Shengjing Hospital of China Medical University 85024, Shenyang, China; Cinvestav-IPN, Mexico City, Mexico

**Keywords:** *Mycoplasma pneumoniae *pneumonia, sputum, mucin, fiberbronchoscopy, bronchoalveolar lavage fluid

## Abstract

To investigate the predictive value of mucin 5AC (MUC5AC) levels in the sputum of children with *Mycoplasma pneumoniae* pneumonia (MPP) treated with fiberbronchoscopy. Children with lobar MPP who were hospitalized in the pediatric pulmonary department of Shengjing Hospital of China Medical University from October 2022 to September 2023 were enrolled. The expression of MUC5AC in sputum was detected by enzyme-linked immunosorbent assay (ELISA). Fiberbronchoalveolar lavage fluid (BALF) was collected, and MUC5AC and epidermal growth factor (EGF) levels in BALF were detected by ELISA, followed by retrospective analysis of the clinical and laboratory test data from each group. The risk factors for fiberbronchoscopy treatment in children with lobar MPP were analyzed by multivariate logistic regression, a nomogram model created, and a receiver operating characteristic curve drawn. Multivariate logistic regression analysis revealed that increased MUC5AC in sputum, lactate dehydrogenase (LDH), IgM levels in serum, and neutrophil-to-lymphocyte ratio (NLR) were risk factors for requiring fiberbronchoscopy treatment in children with lobar MPP (*P* < 0.05); the MUC5AC level in sputum had the highest predictive value (AUC = 0.806). The level of MUC5AC in sputum positively correlated with the levels of LDH, C-reactive protein (CRP), D-dimer, and NLR in serum, and the level of MUC5AC in sputum correlated with the levels of MUC5AC and EGF in BALF in each group. Increased sputum MUC5AC levels, and serum LDH, IgM, and NLR in children with lobar MPP are risk factors for requiring fiberbronchoscopy treatment, and sputum MUC5AC level has superior predictive value to predict the necessity of fiberbronchoscopy treatment to remove a mucus plug.

**IMPORTANCE:**

*Mycoplasma pneumoniae* is a common pathogen of pneumonia in children, and some severely affected children are accompanied by mucus plugs. The level of MUC5AC has important predictive value for mucus hypersecretion. To develop a means of predicting airway mucus secretion in children as early as possible when fiberbronchoscopy could be used to clear airway mucus plugs and reduce sequelae, this article analyzed the risk factors for fiberbronchoscopy treatment in children with lobar *M. pneumoniae* pneumonia (MPP). We identified that the level of MUC5AC in sputum positively correlated with the levels of Lactate dehydrogenase (LDH), C-reactive protein (CRP), D-dimer, and neutrophil-to-lymphocyte ratio (NLR) in serum and the levels of MUC5AC and EGF in BALF of the children with lobar MPP. Increased sputum MUC5AC levels, and serum LDH, IgM, and NLR in children with lobar MPP are risk factors for requiring fiberbronchoscopy treatment, and sputum MUC5AC level has superior predictive value to predict the necessity of fiberbronchoscopy treatment to remove a mucus plug.

## INTRODUCTION

*Mycoplasma pneumoniae* (MP) is one of the most common causes of community-acquired pneumonia in children, and in severe cases can lead to severe *Mycoplasma pneumoniae* pneumonia (SMPP). At present, the first-line drugs to treat MPP in children are macrolide antibiotics, which are efficacious and safe for children with MPP. However, the emergence of drug-resistant MP and the incidence of inflammatory storm caused by multiple cytokines (1, 2) have led to increasing numbers of children suffering from refractory *Mycoplasma pneumoniae* pneumonia (RMPP) and SMPP, most often accompanied by lung consolidation of various degrees (3, 4). Within severe cases, RMPP leads to bronchiolitis obliterans (5, 6). With the investigation into the pathogenesis of MPP and increasing application of fiberbronchoscopy, airway mucus plugs and plastic bronchitis have been recognized as important factors in children with MPP (7–9). Airway mucus hypersecretion is an important mechanism leading to airway mucus plug formation, and mucin is prominent in airway mucus hypersecretion. Mucus and its related components play an important role in the prediction of respiratory diseases (10–12). To reduce MPP complications and sequelae, inhibition of airway mucus hypersecretion and timely removal of mucus plugs have become a key investigative and therapeutic focus.

Mucin, of which many subtypes exist (13), is an important component of airway mucus and is mainly secreted by goblet cells, protecting and lubricating the airway epithelium. MUC5AC plays an important role in the hypersecretion of mucus in airway diseases, and airway epithelium goblet cell hyperplasia and hypersecretion can lead to its overexpression (14–16). Hypersecretion of airway mucus in children with MPP can lead to consolidation, atelectasis, pleural effusion, and plastic bronchitis, aggravating and prolonging the course of the disease, which may cause a poor prognosis (12, 17). Macrolide and glucocorticoid therapy are not effective, and bronchial lavage by fiberbronchoscopy is often needed to remove mucus plugs and relieve airway obstruction. The expression level of MUC5AC was found to be increased in bronchoalveolar lavage fluid (BALF) in children with MPP (14, 18, 19). Therefore, MUC5AC levels have an important value in predicting mucous hypersecretion in children with MPP. At present, the mechanism for excessive MUC5AC secretion induced by MP remains unclear. Most studies have found that MUC5AC secretion is regulated predominantly by the epidermal growth factor receptor (EGFR) signaling pathway (20). Epidermal growth factor (EGF) is a small peptide, which binds to its receptor, EGFR, inducing it to form a dimer, leading to the inhibition of the expression of FOXA2, a major transcriptional inhibitor of mucin biosynthesis, inducing MUC5AC overexpression (21). Bronchial mucus plug formation in RMPP and SMPP is related to a variety of inflammatory mediators (12, 22), which can cause severe airway mucosal injury, impacting ciliary clearance function and epithelial cell exfoliation, and ultimately developing mucus plugs that block the airway. Lactate dehydrogenase (LDH), C-reactive protein (CRP), and D-dimer are the most common indicators of inflammation, are useful for early prediction of RMPP and SMPP, and are also an important indicator of the need for systemic glucocorticoid treatment of children with MPP (23), which may be associated with MUC5AC levels.

MUC5AC has been shown to be involved in the formation of mucous plugs through the detection of MUC5AC levels in BALF (9), but few studies report early indicators to predict mucus hypersecretion. To develop a means of predicting airway mucus secretion in children as early as possible when fiberbronchoscopy could be used to clear airway mucus plugs and reduce sequelae, this study explored the risk factors for the need for fiberbronchoscopy in children with lobar MPP and further analyzed the correlation between sputum MUC5AC levels and serum MUC5AC and EGF.

## MATERIALS AND METHODS

### Participants and inclusion and exclusion criteria

Children older than 1 month and less than 14 years of age who were hospitalized in the pediatric department of Shengjing Hospital of China Medical University with lobar MPP from October 2022 to September 2023 were enrolled. The experimental methods and details of the experimental protocols were approved by the Ethics Committee of China Medical University (NO:20230710; Shenyang, China). All subjects and/or their legal guardians of enrolled children agreed and signed informed consent forms. The treatment of MPP was based on the international guidelines for the diagnosis and treatment of community-acquired pneumonia (24, 25). All the children received standard treatment for MPP, which included supportive care, invasive or non-invasive ventilation, and macrolide antibiotics, as necessary. Children with persistent fever and absent air signs at the site of lobar consolidation identified by lung imaging required treatment with fiberbronchoscopy (26).

**Fig 1:**
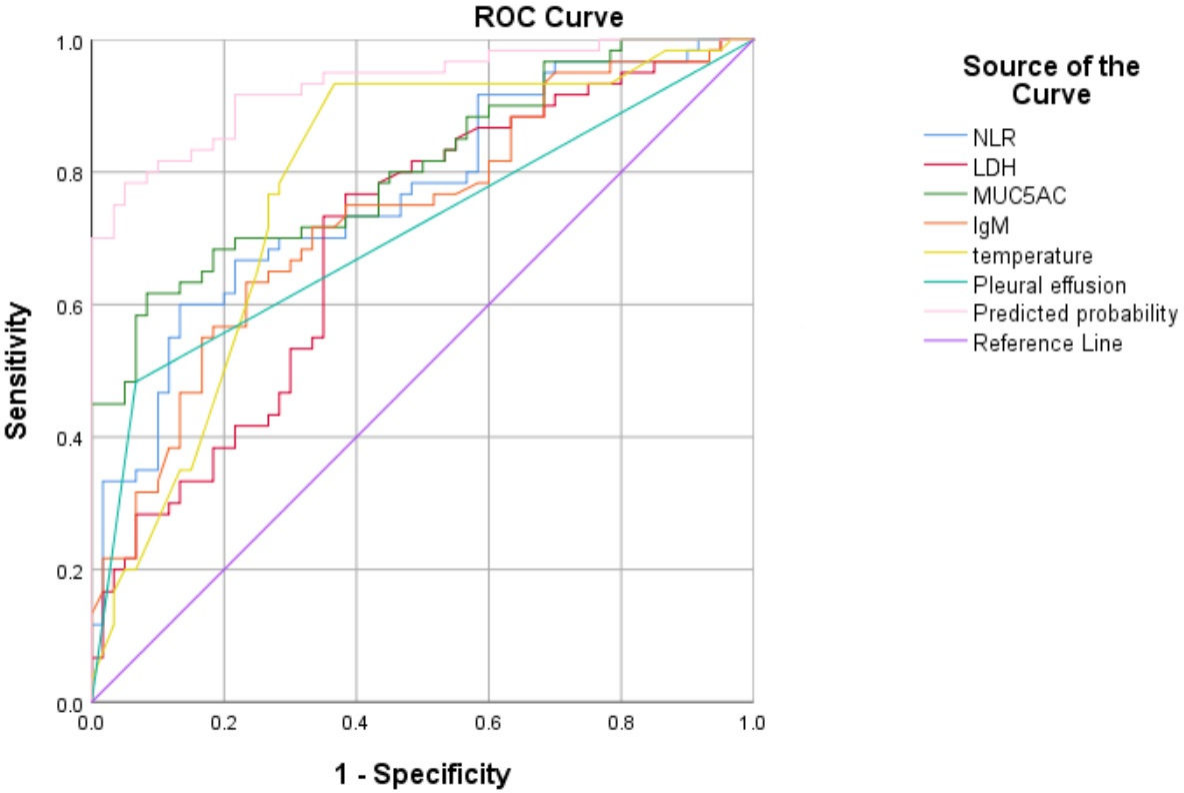
ROC curve for predicting fiberbronchoscopy treatment in children with lobar MPP. The area under the ROC curve was 0.931 (95% CI: 0.886–0.975), indicating excellent predictive accuracy. ROC curve analysis was conducted with sputum MUC5AC level, maximum fever temperature, presence of pleural effusion, serum LDH, NLR, and immunoglobulin IgM as predictors to predict the requirement for fiberbronchoscopy treatment. The corresponding AUC values were 0.806, 0.779, 0.708, 0.697, 0.765, and 0.733, respectively, all of which demonstrated statistical significance.

Children were divided into two groups based on whether they received fiberbronchoscopy treatment. The fiberbronchoscopy treatment group was further divided into the non-mucus plug and mucus plug groups based on the presence of a mucus plug during bronchoalveolar lavage (Fig. 1). Enzyme-linked immunosorbent assay (ELISA) assays were conducted to assess MUC5AC expression in sputum within 24 h after admission. The expression levels of MUC5AC and EGF in BALF of children were analyzed by ELISA. Children with congenital pulmonary dysplasia, airway malformation, congenital immune deficiency, congenital heart disease, malnutrition, bronchiolitis obliterans, bronchial foreign bodies, congenital metabolic diseases, concomitant infection with other pathogens, primary ciliary dyskinesia, cystic fibrosis, asthma, chronic sinusitis, diffuse large B-cell lymphoma, or who had received glucocorticoid therapy or fiberbronchoscopy therapy prior to admission were excluded.

### Study definitions

*Mycoplasma pneumoniae* infection was diagnosed based on positive MP-immunoglobulin M (IgM) antibody detected by serological testing and a positive MP-DNA polymerase chain reaction test from a nasopharyngeal swab sample. MP IgM results with a signal-to-cut off (S/CO) ratio of >1.1 were defined as positive, those with a S/CO of 0.8–1.1 were defined as weak positive, and those with a S/CO < 0.8 were defined as negative. MP antibody (IgM antibody kit; Shenzhen PuRuiKang Biological Technology, Co., Ltd., Shenzhen, China) and MP-DNA screening and identification (MP-DNA detection kit; Shenzhen PuRuiKang Biological Technology, Co., Ltd., Shenzhen, China) were conducted within 24 h of admission. Pneumonia was diagnosed based on symptoms (fever, cough, and/or tachypnea) and clinical evidence of pneumonia (chest recession and/or adventitious sounds on lung auscultation) together with radiographic signs (infiltrates or consolidation).

### Specimen collection and ELISA analysis

Serum *Mycoplasma* antibodies, throat swabs, and sputum samples were collected from all patients within 24 h of admission. Sputum samples using the aseptic negative pressure aspiration method for infants and young children less than 3 years old. In line with the standards for children’s sputum aspiration, a standardized medical sputum aspiration device and disposable sputum aspiration tubes were used. The negative pressure was set between 100 and 200 mmHg, and the procedure was performed by the same nurse. Older children coughed up sputum vigorously after gargling in the morning. Qualified specimens were considered those with a number of polymorphonuclear white blood cells > 25/LP, squamous epithelial cells < 10/LP, and the ratio of multinucleated cells/epithelial cells > 2.5:1. The collection of bronchial lavage fluid specimens is as follows: After administering local anesthesia, insert the fiberoptic bronchoscope into the infected lung segments. Gradually add 37°C sterilized 0.9% sodium chloride through the tracheal biopsy hole, 5 to 10 mL at a time, with a total volume of 15 to 20 mL, not exceeding 30 mL. After each injection, aspirate the liquid at a negative pressure of 100 to 150 mmHg, repeating this three times. Record the volume of the recovered liquid. At least 80% or more should be recovered before analyzing the BALF. All sputum and BALP samples were immediately centrifuged at 4°C for 10 min at 1,000 rpm after collection. The supernatant was then resuspended in 1 mL of 0.9% sodium chloride and stored at −80°C for analysis. ELISA was utilized to assess the sputum MUC5AC levels, and the levels of MUC5AC (MUC5AC ELISA kit, ZK-02633, Shenke, Shanghai, China) and EGF in BALF (EGF ELISA kit, JL10101, Shanghai Jianglai Biotechnology, Co., Ltd., Shanghai, China) following the manufacturer’s instructions.

### Data collection

The requirement for informed consent was waived by the ethics committee. The clinical records and laboratory test data of the children were retrospectively reviewed, and the following data were collected: demographic characteristics (age and sex), medical history (e.g., atopic constitution or history of recurrent respiratory tract infections), and clinical features (including maximum temperature, fever duration, wheezing, extrapulmonary complications, and hypoxemia), pulmonary imaging results (including inflammatory distribution, presence of atelectasis, and pleural effusion), and laboratory test results (white blood cell count, LDH, CRP, and alanine transaminase [ALT]).

### Statistical analysis

SPSS 22.0 statistical software (IBM Corp., Armonk, NY, United States) was used for analysis. Continuous variables were analyzed using the *t*-test or Mann-Whitney U test, while categorical data were examined using the chi-squared test. Logistic regression analysis was conducted to determine risk factors for children with lobar MPP to require bronchoscopy treatment, and R software was used to develop a nomogram model predicting that necessity. Spearman’s rank correlation was utilized to evaluate correlations, and receiver operating characteristic (ROC) curve analysis was performed to assess the optimal predictive threshold of all factors. Results with a *P* value less than 0.05 were considered statistically significant.

## RESULTS

### General information and clinical data of children in the non-fiberbronchoscopy and fiberbronchoscopy treatment groups

We compared the general information between the non-fiberbronchoscopy and fiberbronchoscopy treatment groups in children with lobar MPP and found no statistically significant differences in gender, age, atopic constitution, or history of recurrent infections (*P* > 0.05 for all). However, there were significant differences in clinical features, including maximum temperature, fever duration, wheezing incidence, and presence of extrapulmonary complications, pleural effusion, and atelectasis, as well as percentage of neutrophils, percentage of lymphocytes, neutrophil-to-lymphocyte ratio (NLR), LDH, CRP, D-dimer, ALT, and IgM in serum, and sputum MUC5AC levels (*P* < 0.05 for all). Additionally, there were no differences between the two groups in white blood cell count, Aspartate Aminotransferase (AST), IgG, and creatine kinase MB (CK-MB) (all *P* < 0.05, Table 1).

**TABLE 1:** Clinical characteristics of the children in non-bronchoscopy treatment group and bronchoscopy treatment group^*c*^

Variable	Non bronchoscopy treatment group (*n* = 60)	Bronchoscopy treatment group (*n* = 60)	χ²/Z/T	*P*
General features				
Age (year)	8 (5.00,9.80)	8 (6.80,9.00)	−0.380	0.704
Gender (male/female, n)	31/29	27/31	0.309	0.578
Atopic constitution (n [%])	8 (13.33)	13 (21.67)	1.440	0.230
Repeated infection history (n [%])	2 (3.33)	5 (8.33)	1.365	0.243
Clinical features				
Maximum temperature (°C)	38.9 (38.80,39.50)	39.5 (39.30,40.00)	−5.141	0.000^*b*^
Fever duration (day)	5 (5.00,8.00)	7 (5.00,10.00)	−2.631	0.009^*a*^
Wheezing [n(%)]	9 (15.00)	26 (43.33)	11.657	0.001^*a*^
Extrapulmonary complications (n [%])	8 (13.33)	20 (33.33)	6.708	0.01^*a*^
Hypoxemia (n [%])	0 (0.00)	15 (25.00)	17.777	0.000^*b*^
Pulmonary images				
Pleural effusion (n [%])	4 (6.67)	29 (48.33)	27.490	0.000^*b*^
Atelectasis (n [%])	0 (0.00)	7 (11.67)	7.698	0.006^*a*^
Lobes of inflammation (n [%])				
≤2 lobes	54 (90.00)	22 (36.67)	34.878	0.000^*b*^
≥3 lobes	6 (10.00)	38 (63.33)		
Laboratory test				
Wbc (×10^9^ /L)	9.26 ± 3.38	9.01 ± 3.67	0.373	0.710
Granulocyte (%)	65.20 (51.38,73.63)	71.05 (62.00,81.78)	−2.999	0.003^*a*^
Lymphocyte (%)	25.65 (20.68,39.43)	17.40 (11.83,24.55)	−4.772	0.000^*b*^
NLR	2.13 (1.21,2.76)	4.35 (2.36,7.74)	−4.777	0.000^*b*^
CRP (mg/L)	18.75 (9.70,25.50)	32.00 (22.00,61.00)	−4.536	0.000^*b*^
LDH (U/L)	322.50 (283.00,421.00)	396.50 (349.50,543.50)	−3.732	0.000^*b*^
D-dimer (μg/L)	669 (443.50,860.00)	980 (542.00,3197.00)	−3.256	0.000^*b*^
lgG (g/L)	8.82 (3.90,10.95)	8.75 (7.23,11.12)	−0.823	0.411
lgM (g/L)	1.27 (0.96,1.71)	2.06 (1.24,3.14)	−4.075	0.000^*b*^
ALT (U/L)	18 (12.00,27.50)	31 (17.00,46.50)	−3.288	0.001^*a*^
AST (U/L)	25.50 (22.00,34.00)	27(19.00,44.75)	−0.571	0.568
CK-MB (U/L)	20 (16.00,24.00)	20(15.75,25.00)	−0.119	0.906
Sputum MUC5AC (pg/mL)	6050 ± 2410	9990 ± 4830	12.309	0.001^*a*^

^*a*^ *P<*0.05, vs fiberbronchoscopy treatment group, the difference was statistically significant.

^*b*^ *P* < 0.01, vs fiberbronchoscopy treatment group, significant difference.

^*c*^ CRP, C-reactive protein; PCT, procalcitonin; LDH, lactate dehydrogenase; ALT, alanine transaminase; NLR, neutrophil-to-lymphocyte ratio.

### General information and clinical data of the children in the non-mucus and mucus plug groups

We compared the general information between children with and without mucus plug and found that there were no statistically significant differences between the two groups in gender, age, atopic constitution, history of recurrent infections (all *P* > 0.05). However, there were significant differences in fever duration, incidence of concurrent atelectasis, serum LDH, CRP, and ALT, and sputum MUC5AC levels, as well as MUC5AC and EGF levels in BALF (all *P* < 0.05). There were no differences in maximum temperature, incidence of wheezing, extrapulmonary complications, hypoxemia, pleural effusion, white blood cell count, percentage of neutrophils, percentage of lymphocytes, NLR, D-dimer, AST, IgG, IgM, and CK-MB between the two groups (all *P* > 0.05, Table 2).

**TABLE 2:** Clinical characteristics of the children in the non-mucus and mucus plug groups^*c*^

Variable	Non-mucus plug group (*n* = 30)	Mucus plug group (*n* = 30)	χ²/Z/T	*P*
General features				
Gender (male/female, n)	15/15	12/18	0.606	0.436
Age (year)	7.47 ± 2.53	7.57 ± 2.56	0.027	0.869
Atopic constitution (n [%])	14 (46.67)	17 (56.67)	0.601	0.438
Repeated infection history (n [%])	2 (6.67)	3 (10.00)	0.218	0.64
Clinical features				
Maximum temperature (°C)	39.51 ± 0.80	39.88 ± 0.81	0.744	0.392
Heating time (day)	6.50 (5.00,9.00)	9 (6.75,11.25)	−2.913	0.004^*a*^
Wheezing (n [%])	10 (33.33)	16 (53.33)	2.443	0.118
Extrapulmonary complications (n [%])	8 (26.67)	12 (40.00)	1.200	0.273
Hypoxemia (n [%])	4 (13.33)	11 (36.67)	3.068	0.08
Pulmonary images				
Pleural effusion (n [%])	13 (43.33)	16 (53.33)	0.601	0.438
Atelectasis (n [%])	1 (3.33)	6 (20.00)	0.403	0.044^*a*^
lobes of inflammation (n [%])				
≤2 lobes	20 (66.67)	2(6.67)	11.38	0.001^*a*^
≥3 lobes	10 (33.33)	28 (93.33)		
Laboratory test				
WBC (×10^9^ /L)	9.07 ± 2.92	8.20 ± 4.21	0.211	0.648
Granulocyte (%)	71.10 (62.90,77.90)	70.40 (60.63,84.20)	−0.399	0.69
Lymphocyte (%)	13.45 (11.90,27.60)	17 (13.80,22.50)	−0.828	0.408
NLR	4.43 (2.43,6.20)	3 (2.03,6.79)	−0.934	0.35
CRP (mg/L)	26.05 (19.00,33.00)	40 (22.00,62.00)	−2.152	0.031^*a*^
LDH (U/L)	350.50 (311.25,490.50)	411 (377.50,577.00)	−2.624	0.009^*a*^
DD (μg/L)	344.50 (172.75,1157.00)	745.50 (286.50,1929.50)	−1.39	0.165
lgG (g/L)	9.04 ± 2.86	9.38 ± 2.18	2.284	0.136
lgM (g/L)	2 (1.10,3.14)	1.53 (1.35,2.33)	−0.251	0.802
ALT (U/L)	29.50 (14.75,3.25)	40 (18.75,75.50)	−2.004	0.045^*a*^
AST (U/L)	27.50 (19.75,38.25)	34 (21.75,55.00)	−1.509	0.131
CK-MB (U/L)	20 (17.00,25.25)	19.50 (15.75,22.50)	−0.786	0.432
Sputum MUC5AC (pg/mL)	7750 ± 2870	12240 ± 5360	4.041	0.000^*b*^
BALF MUC5AC (pg/mL)	10150 ± 5160	13970 ± 8830	2.899	0.005^*a*^
BALF EGF (pg/mL)	54.63 (40.90,61.70)	75.88 (54.20,148.20)	−3.075	0.002^*a*^

^*a*^ *P<*0.05, vs fiberbronchoscopy treatment group, the difference was statistically significant.

^*b*^ *P* < 0.01, vs fiberbronchoscopy treatment group, significant difference.

^*c*^ CRP, C-reactive protein; PCT, procalcitonin; LDH, lactate dehydrogenase; ALT, alanine transaminase; NLR, neutrophil-to-lymphocyte ratio; EGF, epidermal growth factor.

### Risk factors for requiring fiberbronchoscopy treatment in children with lobar MPP

The necessity for fiberbronchoscopy treatment in children with lobar MPP was designated as the dependent variable, and the maximum temperature, fever duration, presence of pleural effusion, presence of atelectasis, neutrophil count, lymphocyte count, NLR, the levels of serum CRP, LDH, ALT, D-dimer, and IgM, and sputum MUC5AC levels were considered as the independent variables. All variables were treated as continuous variables. Stepwise regression analysis revealed that the maximum temperature, presence of pleural effusion, NLR, the level of LDH in serum, sputum MUC5AC level, and serum IgM were independent risk factors for requiring fiberbronchoscopy treatment (all *P* < 0.05, Table 3).

**TABLE 3:** Multivariate logistic regression analysis of the risk factors of fiberbronchoscopy treatment in children with lobar MPP^*a*^

Factor	β	SE	Wald χ²	P	OR	95% CI
Maximum temperature (°C)	1.25	0.46	7.23	0.006	3.51	(1.42,8.64）
Pleural effusion (n [%])	1.96	0.93	4.41	0.036	7.06	(1.14,43.79）
NLR	0.52	0.20	6.87	0.009	1.69	(1.14,2.49）
LDH (U/L)	0.02	0.01	9.71	0.002	1.01	(1.01,1.03）
lgM (g/L)	1.14	0.48	5.50	0.019	3.12	(1.21, 8.05)
Sputum MUC5AC (pg/mL)	0.00	0.00	6.83	0.009	1.00	(1.00, 1.00)
Constant	−65.68	20.24	10.53	0.001	0.00	-

^*a*^ *P<0.05, vs fiberbronchoscopy treatment group, the difference was statistically significant.

### ROC curve analysis for predicting need for fiberbronchoscopy treatment in children with Lobar MPP

**Fig 2:**
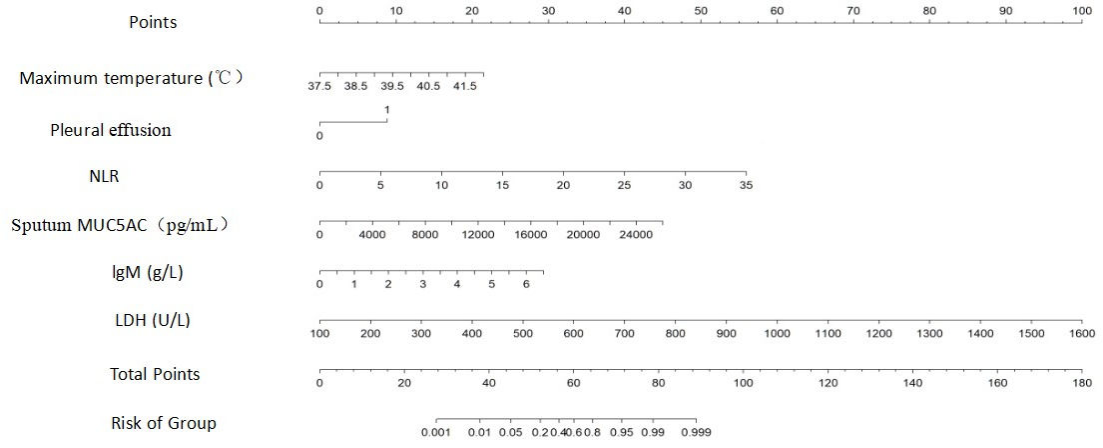
A column chart model for fibrous bronchial lavage in children with lobar MPP The result showed that with every 1°C rise in maximum fever temperature, the score increased by 4.77 points; for every 1% increase in the percentage of pleural effusion, the score increased by 8.86; for one unit increase in NLR, the score increased by 1.60 points; similarly, for every 1,000 pg/mL increase in MUC5AC, the score increased by 1.73 points; every 1 g/L increase in IgM, the score increased by 4.52 points; every 100 U/L surge in LDH, the score escalated by 6.67 points.

The predictive efficacy of the nomogram model for determining the necessity of fiberbronchoscopy treatment in children with lobar MPP was verified using ROC curve analysis. The area under the ROC curve was 0.931 (95% confidence interval [CI]: 0.886–0.975), indicating excellent predictive accuracy. Furthermore, a further ROC curve analysis was conducted using risk factors (sputum MUC5AC level, maximum temperature, presence of pleural effusion, and serum LDH, NLR, and IgM) as predictors to predict risk for requiring fiberbronchoscopy treatment. The corresponding AUC values were 0.806, 0.779, 0.708, 0.697, 0.765, and 0.733, respectively, all of which demonstrated statistical significance. The cutoff value for sputum MUC5AC level was 8,774 pg/mL, for serum LDH 348.5 U/L, for serum IgM 3.39 g/L, for NLR 1.75, for maximum temperature 38.9°C, all of which indicate that children with lobar MPP require fiberbronchoscopy (*P* < 0.05, Table 4; Fig. 2).

**TABLE 4:** Predictive risk of bronchofibroscopy treatment in children with lobar MPP

Factor	AUC	95% CI	Cut off	Youden index	Susceptibility	Specificity
MUC5AC in sputum (pg/mL）	0.81	0.73–0.88	8774.00	0.53	61.70%	91.70%
LDH (U/L）	0.70	0.60–0.79	348.50	0.38	76.70%	61.70%
lgM (g/L）	0.73	0.64–0.82	3.39	0.40	60.00%	86.70%
NLR	0.76	0.68–0.85	1.75	0.47	63.30%	76.70%
Maximum temperature (°C）	0.78	0.69–0.87	38.95	0.57	93.30%	63.30%
Pleural effusion	0.71	0.61–0.80	^*a*^-	0.42	48.30%	93.30%
Predicted Probability (%）	0.93	0.89–0.98	62.36	0.73	78.30%	95.00%

^*a*^ "-" means that no numerical calculation can be performed.

### Nomogram for prediction of the need for fiberbronchoscopy treatment in children with lobar MPP

**Fig 3:**
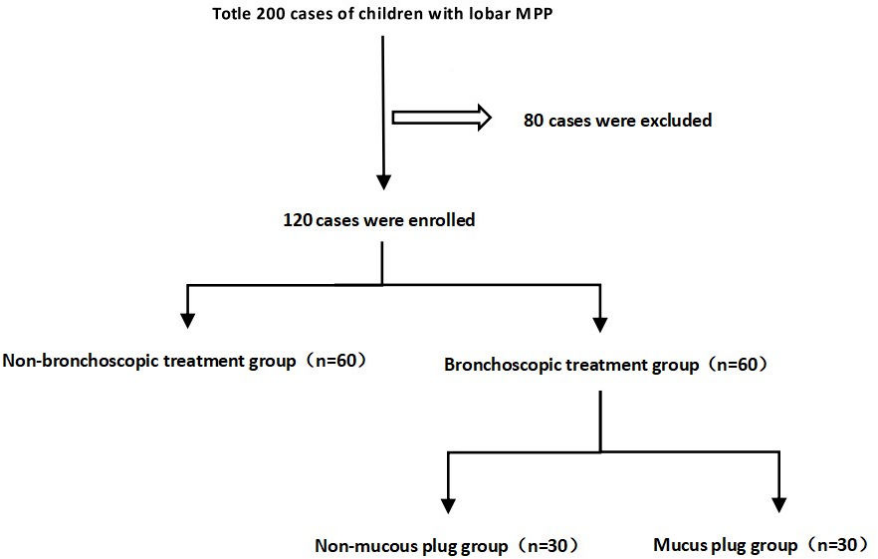
Study enrollment and grouping. A total of 200 children with *Mycoplasma* lobar pneumonia were enrolled; 80 children who did not meet the experimental criteria were excluded according to the exclusion criteria, and the last 120 children participated in the experiment. There were 60 patients treated with fiberoptic bronchoscopy and 60 patients treated without fiberbronchoscopy treatment. Fiberbronchoscopy treatment group was divided into 30 patients with mucus plug and 30 patients with non-mucus plug according to whether there was mucus plug under the microscope.

All these risk factors were put into R software to construct a nomogram for predicting the need for fiberbronchoscopy treatment in children with lobar MPP. The result showed that with every 1°C rise in maximum temperature, the score increased by 4.77 points; with every 1% increase in the percentage presence of pleural effusion, it increased by 8.86; and with each unit increase in NLR, it increased by 1.60 points. Similarly, with every 1,000 pg/mL increase in sputum MUC5AC level, the score increased by 1.73 points; with every 1 g/L increase in IgM, it increased by 4.52 points; and with every 100 U/L increase in LDH, it increased by 6.67 points (Fig. 3).

### Correlation between the level of MUC5AC in sputum and levels of MUC5AC in BALF, and LDH, CRP, D-dimer, and NLR in serum

Spearman’s rank correlation analysis was used to assess the correlation between the sputum MUC5AC level and EFG in BALF in the fiberbronchoscopy group. There was a positive correlation between sputum MUC5AC level and MUC5AC and EGF levels in BALF (r = 0.613 and 0.358, *P* < 0.05). There was also a positive correlation between the level of MUC5AC in sputum and LDH in serum and with NLR (r = 0.816, 0.436, *P* < 0.05), while no correlation was found between the level of MUC5AC in sputum and serum IgM (*P* > 0.05) (Table 5).

**TABLE 5:** Correlation between the level of MUC5AC in sputum and levels of MUC5AC and EFG in BALF, LDH, CRP, D-dimer, and NLR in serum

	MUC5AC in sputum	
	R	*P*
MUC5AC in BALF	0.613	*P* ＜ 0.05
EFG in BALF	0.358	*P* ＜ 0.05
LDH in serum	0.816	*P* ＜ 0.05
NLR in serum	0.436	*P* ＜ 0.05
CRP in serum	0.563	*P* ＜ 0.05
DD in serum	0.358	*P* ＜ 0.05

## DISCUSSION

Airway mucus is mainly composed of mucin, electrolyte, and water; mucin is one of the important components of airway mucus. Mucins are encoded by the MUC gene and are widely distributed on the mucosal epithelial surface of various tissues (27, 28). In physiological conditions, the content of MUC5AC is small, but in pathological conditions, MUC5AC can account for 95% of the total mucin secretion of airway epithelia, and its abnormal secretion often represents airway diseases. Therefore, studies on the high secretion of airway mucus tend to focus on MUC5AC (29, 30). A number of studies have found that high expression of MUC5AC is associated with a variety of inflammatory respiratory diseases, such as acute viral or bacterial infections and chronic airway inflammation (31, 32). *M. pneumoniae* infection in children has a specific epidemic season every year, especially from September to December, reaching a peak every 2–3 years in the north of China. A tendency to break out every few years was also observed (33). In 2023, outbreaks of MPP have been reported in various parts of China. MPP tends to occur in densely populated environments, such as kindergartens and schools, and is the primary pathogen of community-acquired pneumonia in hospitalized children (34–36). MP is one of the most common and important pathogens of community-acquired pneumonia. Numerous studies have found that MP infection can induce abnormal secretion of MUC5AC in goblet cells, resulting in hypersecretion of airway mucus, causing partial or complete ventilation dysfunction and dyspnea in children, and affecting the development and prognosis of MPP (19). A number of studies have confirmed that the expression level of MUC5AC in BALF in children with MPP is closely related to the severity of the disease and the formation of airway mucus plugs. High expression of MUC5AC in BALF is mostly in the airway of the children with RMPP, and the pneumonia progresses rapidly. The fever time and hospitalization time are long, complicated with lung consolidation, pleural effusion, and pulmonary atelectasis (23).

Many studies have detected the expression level of MUC5AC in BALF and confirmed that MUC5AC is significantly increased in children with MPP accompanied by mucous plugs, and its content is positively correlated with the degree of airway mucus viscosity (31). Some scholars have shown that the increased expression of MUC5AC in BALF is a risk factor for MPP complicated with plastic bronchitis (12). In addition, the increased MUC5AC content in BALF and sputum indicates the formation of airway mucus plugs, but MUC5AC in BALF cannot predict the degree of mucous viscosity early. This study investigated the correlation between the expression level of MUC5AC in sputum and the content of MUC5AC in BALF, in order to predict the mucus hypersecretion in BALF by detecting the changes in the expression level of MUC5AC in sputum. The results of this study showed that the expression levels of MUC5AC in the sputum of children in the fiberbronchoscopy treatment group were significantly higher than that in the non-fiberbronchoscopy tratment group. The MUC5AC content in sputum and in BALF of the children in the mucus plug group was significantly higher than that in the non-mucus plug group, and MUC5AC in sputum was positively correlated with MUC5AC level in BALF. Therefore, we could predict the degree of mucus viscosity in the respiratory tract of children with different severity by early detection of MUC5AC content in sputum. To improve the prognosis of pneumonia and avoid sequelae by inhibiting the over-expression of mucin in the respiratory tract and early lavatory with fiber bronchoscopy to clear mucus plug so as to relieve airway obstruction. In recent years, it has been reported that MUC5AC secretion is mainly regulated by the EGFR signaling pathway (18), and EGF is a key factor inducing MUC5AC overexpression. This study found that EGF level in BALF of children with the mucus plug group was significantly higher than that in the non-mucus plug group, and there was a positive correlation between MUC5AC and EGF in BALF, but the mechanism needs further study.

After MP infects the host, the overactive immune activation caused by the cytokine-mediated inflammatory response in the host plays an important role in the progression of the disease, and the production of inflammatory factors is related to the severity of MPP (37). Therefore, the use of relevant biological predictors for early identification of severe patients and timely treatment will help reduce the risk of poor prognosis. In this study, multivariate logistic analysis showed that high fever, accompanied by pleural effusion, increased levels of NLR, LDH, immunoglobulin lgM in serum, and MUC5AC in sputum are independent risk factors for fiberbronchoscopy treatment in children with lobar MPP, which was consistent with previous research (22, 23). We constructed a nomogram model to predict the risk of fiberbronchoscopy treatment in children with lobar MPP. The results showed that the nomogram prediction model constructed in this study had high consistency and good prediction effect, which could help clinicians evaluate the risk of fiberbronchoscopy treatment in children with lobar MPP of different severity, so as to take different treatment measures. In addition, ROC curves were plotted for different risk factors in this study, and the results showed that the increased levels of MUC5AC in sputum, LDH and immunoglobulin lgM in serum, NLR, maximum temperature, and the occurrence of pleural effusion were all effective predictors for early fiberbronchoscopy treatment for MPP children with lobar pneumonia. When MUC5AC level in sputum is more than 8,774 pg/mL, the LDH level in serum is more than 348 U/L, immunoglobulin IgM level is more than 3.39 g/L, NLR is more than 1.75, maximum temperature is more than 38.9°C, and the presence of pleural effusion, which will indicate the children need to be treated with fiberbronchoscopy. At the same time, all the risk factors were input into R software to construct a nomogram model for predicting fiberbronchoscopy treatment in children with lobar MPP. The level of LDH and higher fever have been reported before, but MUC5AC levels in sputum were first reported as a predictor of risk for fiberbronchoscopy treatment.

In addition, relevant studies have found that airway mucus hypersecretion is not a random phenomenon, but an indicator of immune response, which is related to a variety of inflammatory factors (12, 22). LDH, as the most common and economical indicator in practice, has significant reference significance for early prediction of RMPP and SMPP (38, 39). Moreover, the nomogram model in this study found that LDH had certain value in predicting the risk of fiberbronchoscopy treatment of MPP. This study further analyzed the correlation between MUC5AC in sputum and LDH in serum. The results showed that the level of MUC5AC in sputum was positively correlated with LDH in serum. Therefore, we can predict the serum LDH level by detecting the expression level of MUC5AC in sputum, so as to predict the severity of pneumonia in children earlier.

In summary, the hypersecretion of airway mucus is involved in the pathogenesis of MPP, and the expression level of MUC5AC is closely related to the formation of mucus plug and the severity of the disease. The results of this study showed that there was a significant positive correlation between MUC5AC in sputum and MUC5AC in BALF. MUC5AC expression level in sputum can help predict the time of fiberbronchoscopy treatment for SMPP. At the same time, the increased levels of MUC5AC in sputum, LDH and immunoglobulin lgM in serum, and NLR of the children with lobar MPP were risk factors for fiberbronchoscopy treatment. Additionally, the level of MUC5AC in sputum was closely related to the content of LDH in serum. Therefore, the level of MUC5AC in sputum can replace LDH in serum to early predict the severity of children and guide fiberbronchoscopy treatment to remove the mucus plug in order to reduce complications. However, this study still has many limitations. In this study, only 60 cases with REPP underwent fiberoptic bronchoscopy, so multi-center and large-sample clinical trials are still needed to further confirm the experimental results. In addition, plastic bronchitis does not exist in all children with *Mycoplasma* infection and does not have a high incidence rate every year, only during the *Mycoplasma* epidemic season. It can be seen that it is related to the epidemic trend of *Mycoplasma* and the genetic variations of the pathogen, so the correlation between the two factors needs to be further studied.

## Data Availability

The data sets used and/or analyzed in this study are available on request from the corresponding author or can be accessed from the following link: https://www.jianguoyun.com/p/DalQG1kQ65arCxjWzf8FIAA
