# Correction for Jian et al., “The predictive value of MUC5AC levels in the sputum of children with *Mycoplasma pneumoniae* pneumonia treated with fiberbronchoscopy”

**DOI:** 10.1128/spectrum.03919-25

**Published:** 2025-12-11

**Authors:** Xiao-qing Jian, Xiao-yan Wang, Miao Li

## AUTHOR CORRECTION

Volume 13, no. 8, e00103-25, 2025, https://doi.org/10.1128/spectrum.00103-25. Page 1, byline: The author order should be as shown in this correction.

